# Supplementary material for: Combined effects of gliding-arc plasma and C-phycocyanin on antioxidant activity and shelf-life extension of rainbow trout (Oncorhynchus mykiss) fillets
Source: PLoS One. 2025 Nov 20;20(11):e0336896. doi: 10.1371/journal.pone.0336896 (PMC12633869; doi:10.1371/journal.pone.0336896)
Supplement: S1 Fig — The absorbance of the purified C-phycocyanin (PCP) was measured across the wavelength range of 260–800 nm, showing a distinct characteristic absorption peak at 621.9 nm, confirming the presence of highly pure PCP. The pronounced absorption peak at 621.9 nm indicates the presence of the chromophore phycocyanobilin and verifies the successful purification of the PCP. (DOCX) [file pone.0336896.s001.docx]

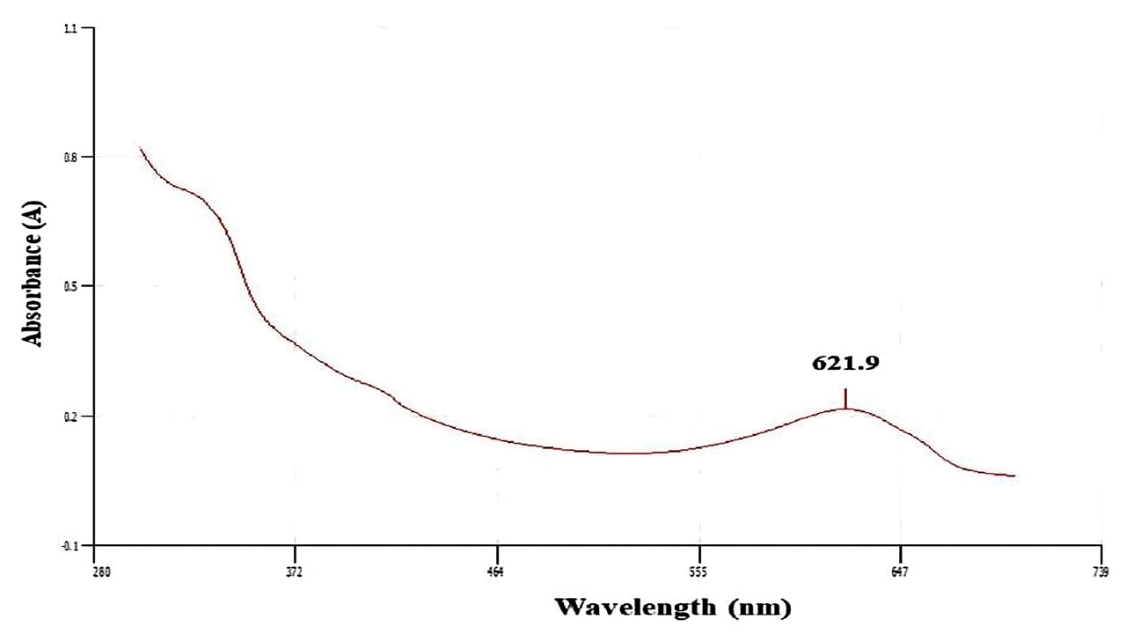


Fig. S1. Absorption spectrum of purified C-phycocyanin pigment extracted from cyanobacterial biomass. The absorbance of the purified C-phycocyanin (PCP) was measured across the wavelength range of 260–800 nm, showing a distinct characteristic absorption peak at 621.9 nm, confirming the presence of highly pure PCP. The pronounced absorption peak at 621.9 nm indicates the presence of the chromophore phycocyanobilin and verifies the successful purification of the PCP.
