# Supplementary material for: Combined effects of gliding-arc plasma and C-phycocyanin on antioxidant activity and shelf-life extension of rainbow trout (Oncorhynchus mykiss) fillets
Source: PLoS One. 2025 Nov 20;20(11):e0336896. doi: 10.1371/journal.pone.0336896 (PMC12633869; doi:10.1371/journal.pone.0336896)
Supplement: S2 Fig — Sensory evaluation was performed on days 1, 3, 6, 9, 12, 15, and 18. Treatment groups: C, PC-P, P2-PC, P5-PC, P2 + PC, and P5 + PC.Values represent mean ± SEM (n = 3). Significant differences were determined by one-way ANOVA followed by Tukey’s test (p < 0.05). Different lowercase letters indicate significant differences among treatments within the same day, and uppercase letters among storage days within the same treatment. (DOCX) [file pone.0336896.s002.docx]

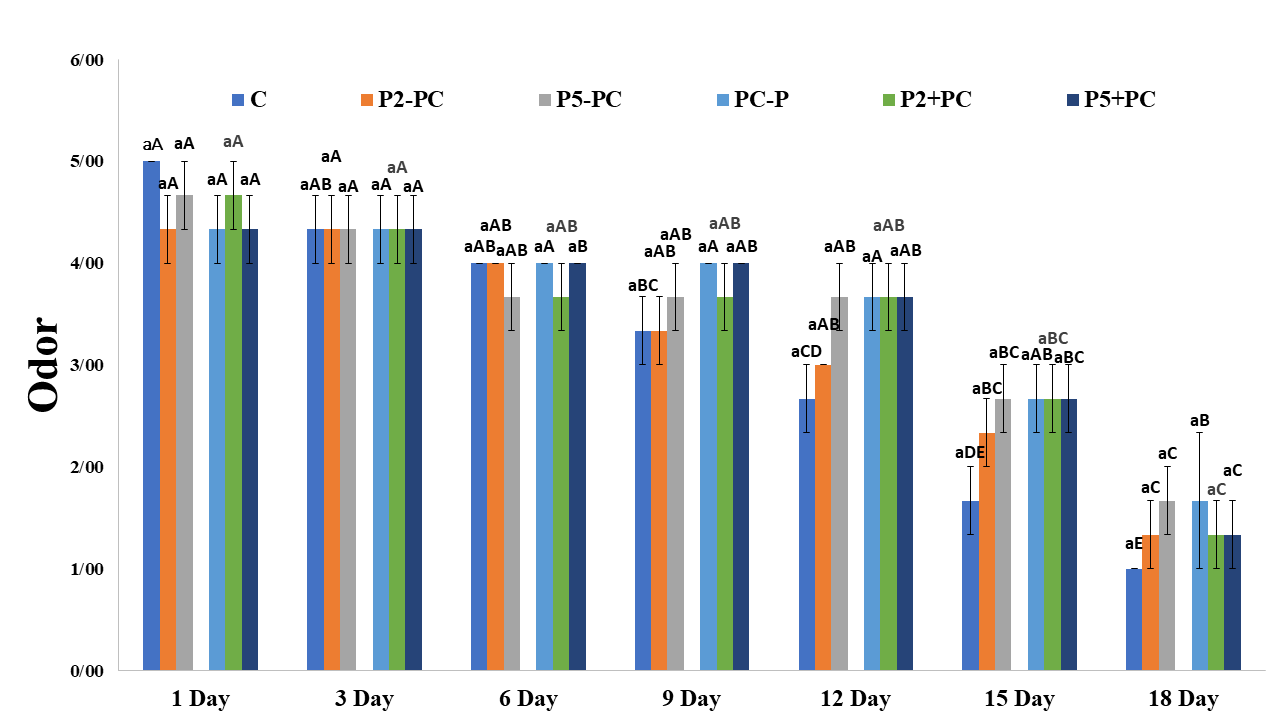


Fig. S2. **Results of odor of rainbow trout fillets during 18 days of refrigerated storage (4 °C).** Sensory evaluation was performed on days 1, 3, 6, 9, 12, 15, and 18. Treatment groups: C, PC-P, P2-PC, P5-PC, P2+PC, and P5+PC.Values represent mean ± SEM (n = 3). Significant differences were determined by one-way ANOVA followed by Tukey’s test (p < 0.05). Different lowercase letters indicate significant differences among treatments within the same day, and uppercase letters among storage days within the same treatment.
