# Supplementary material for: Combined effects of gliding-arc plasma and C-phycocyanin on antioxidant activity and shelf-life extension of rainbow trout (Oncorhynchus mykiss) fillets
Source: PLoS One. 2025 Nov 20;20(11):e0336896. doi: 10.1371/journal.pone.0336896 (PMC12633869; doi:10.1371/journal.pone.0336896)
Supplement: S1 Table — C: control sample (without plasma treatment and phycocyanin pigment); PC-P: sample treated with phycocyanin pigment but without plasma; P2-PC: plasma-treated sample for 2 min without phycocyanin pigment; P5-PC: plasma-treated sample for 5 min without phycocyanin pigment; P2 + PC: plasma-treated sample for 2 min with phycocyanin pigment; P5 + PC: plasma-treated sample for 5 min with phycocyanin pigment. Different small and capital letters indicate significant differences in the columns and rows, respectively (p < 0.05). All data are expressed as mean ± SEM (n = 3). Data were analyzed using one-way ANOVA followed by Tukey’s post hoc test (p < 0.05). (DOCX) [file pone.0336896.s005.docx]

**Table S1.** Mean PTC of *Oncorhynchus mykiss* fillets treated with GAP and PCP during storage at 4°C for 18 days.

| **PTC** | **Day1** | **Day3** | **Day6** | **Day9** | **Day12** | **Day15** | **Day18** |
| --- | --- | --- | --- | --- | --- | --- | --- |
| **C** | 3.32±0.0063(a)(A) | 5.38±0.0046(a)(B) | 6.38±0.0089(a)(C) | 7.35±0.0053(a)(D) | 8.35±0.0062(a)(E) | 9.21±0.0054(a)(F) | 10.39±0.0047(a)(G) |
| **P2-PC** | 3.14±0.0080(b)(A) | 4.98±0.0198(b)(B) | 5.95±0.0192(b)(C) | 6.39±0.0027(b)(D) | 7.38±0.0067(b)(E) | 8.32±0.0105(b)(F) | 9.29±0.0113(b)(G) |
| **P5-PC** | 2.95±0.0168(c)(A) | 4.34±0.0102(c)(B) | 5.18±0.0060(c)(C) | 6.13±0.0104(c)(D) | 7.03±0.0046(c)(E) | 7.81±0.0178(c)(F) | 8.70±0.0227(c)(G) |
| **PC-P** | 3.14±0.0173(b)(A) | 5.13±0.0060(d)(B) | 6.09±0.0082(d)(C) | 6.68±0.0380(d)(D) | 7.80±0.0145(d)(E) | 8.86±0.0122(d)(F) | 9.77±0.0151(d)(G) |
| **P2+PC** | 2.97±0.0083(c)(A) | 3.96±0.0183(e)(B) | 4.79±0.0181(e)(C) | 5.20±0.0072(e)(D) | 6.30±0.0058(e)(E) | 7.03±0.0074(e)(F) | 7.97±0.0085(e)(G) |
| **P5+PC** | 2.70±0.0078(d)(A) | 3.56±0.0411(f)(B) | 4.21±0.0489(f)(C) | 4.63±0.0090(f)(D) | 5.84±0.0147(f)(E) | 6.42±0.0044(f)(F) | 7.17±0.0059(f)(G) |

C: control sample (without plasma treatment and phycocyanin pigment); PC-P: sample treated with phycocyanin pigment but without plasma; P2-PC: plasma-treated sample for 2 min without phycocyanin pigment; P5-PC: plasma-treated sample for 5 min without phycocyanin pigment; P2+PC: plasma-treated sample for 2 min with phycocyanin pigment; P5+PC: plasma-treated sample for 5 min with phycocyanin pigment. Different small and capital letters indicate significant differences in the columns and rows, respectively (p < 0.05). All data are expressed as mean ± SEM (n = 3). Data were analyzed using one-way ANOVA followed by Tukey’s post hoc test (p < 0.05).
