# Supplementary material for: Combined effects of gliding-arc plasma and C-phycocyanin on antioxidant activity and shelf-life extension of rainbow trout (Oncorhynchus mykiss) fillets
Source: PLoS One. 2025 Nov 20;20(11):e0336896. doi: 10.1371/journal.pone.0336896 (PMC12633869; doi:10.1371/journal.pone.0336896)
Supplement: S2 Table — C: control sample (without plasma treatment and phycocyanin pigment); PC-P: sample treated with phycocyanin pigment but without plasma; P2-PC: plasma-treated sample for 2 min without phycocyanin pigment; P5-PC: plasma-treated sample for 5 min without phycocyanin pigment; P2 + PC: plasma-treated sample for 2 min with phycocyanin pigment; P5 + PC: plasma-treated sample for 5 min with phycocyanin pigment. Different small and capital letters indicate significant differences in the columns and rows, respectively (p < 0.05). All data are expressed as mean ± SEM (n = 3). Data were analyzed using one-way ANOVA followed by Tukey’s post hoc test (p < 0.05). (DOCX) [file pone.0336896.s006.docx]

**Table S2.** Mean Acidity of *Oncorhynchus mykiss* fillets treated with GAP and PCP during storage at 4°C for 18 days.

| **Acidity** | **Day1** | **Day3** | **Day6** | **Day9** | **Day12** | **Day15** | **Day18** |
| --- | --- | --- | --- | --- | --- | --- | --- |
| **C** | 0.13±0.0010(a)(A) | 0.15±0.0005(a)(B) | 0.16±0.0005(a)(C) | 0.17±0.0008(a)(D) | 0.18±0.0005(a)(E) | 0.19±0.0005(a)(F) | 0.23±0.0021(a)(G) |
| **P2-PC** | 0.13±0.0006(a)(A) | 0.14±0.0008(b)(B) | 0.15±0.0005(b)(C) | 0.17±0.0003(a)(D) | 0.17±0.0003(b)(E) | 0.18±0.0005(b)(F) | 0.20±0.0008(b)(G) |
| **P5-PC** | 0.13±0.0000(a)(A) | 0.14±0.0005(cd)(B) | 0.15±0.0008(c)(C) | 0.16±0.0005(b)(D) | 0.17±0.0016(c)(E) | 0.18±0.0005(c)(F) | 0.19±0.0008(c)(G) |
| **PC-P** | 0.13±0.0006(a)(A) | 0.14±0.0005(bc)(B) | 0.15±0.0008(b)(C) | 0.16±0.0005(c)(D) | 0.17±0.0008(b)(E) | 0.18±0.0010(c)(F) | 0.20±0.0005(b)(G) |
| **P2+PC** | 0.13±0.0003(a)(A) | 0.14±0.0008(d)(B) | 0.14±0.0013(c)(C) | 0.15±0.0008(d)(D) | 0.16±0.0006(d)(E) | 0.17±0.0005(d)(F) | 0.18±0.0008(d)(G) |
| **P5+PC** | 0.13±0.0003(a)(A) | 0.13±0.0003(e)(B) | 0.14±0.0005(d)(C) | 0.15±0.0008(e)(D) | 0.15±0.0005(e)(E) | 0.16±0.0008(e)(F) | 0.17±0.0008(e)(G) |

C: control sample (without plasma treatment and phycocyanin pigment); PC-P: sample treated with phycocyanin pigment but without plasma; P2-PC: plasma-treated sample for 2 min without phycocyanin pigment; P5-PC: plasma-treated sample for 5 min without phycocyanin pigment; P2+PC: plasma-treated sample for 2 min with phycocyanin pigment; P5+PC: plasma-treated sample for 5 min with phycocyanin pigment. Different small and capital letters indicate significant differences in the columns and rows, respectively (p < 0.05). All data are expressed as mean ± SEM (n = 3). Data were analyzed using one-way ANOVA followed by Tukey’s post hoc test (p < 0.05).
