# Supplementary material for: Combined effects of gliding-arc plasma and C-phycocyanin on antioxidant activity and shelf-life extension of rainbow trout (Oncorhynchus mykiss) fillets
Source: PLoS One. 2025 Nov 20;20(11):e0336896. doi: 10.1371/journal.pone.0336896 (PMC12633869; doi:10.1371/journal.pone.0336896)
Supplement: S3 Table — C: control sample (without plasma treatment and phycocyanin pigment); PC-P: sample treated with phycocyanin pigment but without plasma; P2-PC: plasma-treated sample for 2 min without phycocyanin pigment; P5-PC: plasma-treated sample for 5 min without phycocyanin pigment; P2 + PC: plasma-treated sample for 2 min with phycocyanin pigment; P5 + PC: plasma-treated sample for 5 min with phycocyanin pigment. Different small and capital letters indicate significant differences in the columns and rows, respectively (p < 0.05). All data are expressed as mean ± SEM (n = 3). Data were analyzed using one-way ANOVA followed by Tukey’s post hoc test (p < 0.05). (DOCX) [file pone.0336896.s007.docx]

**Table S3.** Mean TBARS of *Oncorhynchus mykiss* fillets treated with GAP and PCP during storage at 4°C for 18 days.

| **TBARS** | **Day1** | **Day3** | **Day6** | **Day9** | **Day12** | **Day15** | **Day18** |
| --- | --- | --- | --- | --- | --- | --- | --- |
| **C** | 0.21±0.0000(a)(A) | 0.36±0.0023(a)(B) | 0.46±0.0030(a)(C) | 0.60±0.0041(a)(D) | 0.71±0.0030(a)(E) | 0.77±0.0060(a)(F) | 0.89±0.0059(a)(G) |
| **P2-PC** | 0.20±0.0011(ab)(A) | 0.26±0.0030(b)(B) | 0.34±0.0020(b)(C) | 0.46±0.0020(b)(D) | 0.57±0.0030(b)(E) | 0.55±0.0023(b)(F) | 0.67±0.0020(b)(G) |
| **P5-PC** | 0.19±0.0000(b)(A) | 0.24±0.0030(c)(B) | 0.31±0.0020(c)(C) | 0.42±0.0023(c)(D) | 0.52±0.0082(c)(E) | 0.50±0.0041(c)(F) | 0.60±0.0011(c)(G) |
| **PC-P** | 0.17±0.0020(c)(A) | 0.32±0.0020(d)(B) | 0.42±0.0030(d)(C) | 0.56±0.0020(d)(D) | 0.65±0.0030(d)(E) | 0.69±0.0030(d)(F) | 0.79.±0.0020(d)(G) |
| **P2+PC** | 0.17±0.0020(cd)(A) | 0.24±0.0030(c)(B) | 0.30±0.0020(c)(C) | 0.42±0.0020(c)(D) | 0.51±0.0030(c)(E) | 0.46±0.0050(c)(F) | 0.56±0.0030(c)(G) |
| **P5+PC** | 0.16±0.0039(d)(A) | 0.19±0.0020(e)(B) | 0.27±0.0020(e)(C) | 0.38±0.0020(e)(D) | 0.46±0.0030(e)(E) | 0.43±0.0030(e)(F) | 0.51±0.0050(e)(G) |

C: control sample (without plasma treatment and phycocyanin pigment); PC-P: sample treated with phycocyanin pigment but without plasma; P2-PC: plasma-treated sample for 2 min without phycocyanin pigment; P5-PC: plasma-treated sample for 5 min without phycocyanin pigment; P2+PC: plasma-treated sample for 2 min with phycocyanin pigment; P5+PC: plasma-treated sample for 5 min with phycocyanin pigment. Different small and capital letters indicate significant differences in the columns and rows, respectively (p < 0.05). All data are expressed as mean ± SEM (n = 3). Data were analyzed using one-way ANOVA followed by Tukey’s post hoc test (p < 0.05).
