# Supplementary material for: Combined effects of gliding-arc plasma and C-phycocyanin on antioxidant activity and shelf-life extension of rainbow trout (Oncorhynchus mykiss) fillets
Source: PLoS One. 2025 Nov 20;20(11):e0336896. doi: 10.1371/journal.pone.0336896 (PMC12633869; doi:10.1371/journal.pone.0336896)
Supplement: S5 Table — C: control sample (without plasma treatment and phycocyanin pigment); PC-P: sample treated with phycocyanin pigment but without plasma; P2-PC: plasma-treated sample for 2 min without phycocyanin pigment; P5-PC: plasma-treated sample for 5 min without phycocyanin pigment; P2 + PC: plasma-treated sample for 2 min with phycocyanin pigment; P5 + PC: plasma-treated sample for 5 min with phycocyanin pigment. Different small and capital letters indicate significant differences in the columns and rows, respectively (p < 0.05). All data are expressed as mean ± SEM (n = 3). Data were analyzed using one-way ANOVA followed by Tukey’s post hoc test (p < 0.05). (DOCX) [file pone.0336896.s009.docx]

**Table S5.** Mean a* of *Oncorhynchus mykiss* fillets treated with GAP and PCP during storage at 4°C for 18 days.

| **a*** | **Day1** | **Day3** | **Day6** | **Day9** | **Day12** | **Day15** | **Day18** |
| --- | --- | --- | --- | --- | --- | --- | --- |
| **C** | 2.26±0.0586(a)(A) | 2.78±0.0837(a)(A) | 3.33±0.1629(a)(B) | 4.02±0.2291(a)(C) | 5.09±0.0233(a)(D) | 7.33±0.1004(a)(E) | 9.18±0.0233(a)(F) |
| **C2-PC** | 3.51±0.1278(b)(A) | 3.76±0.0624(b)(AB) | 4.13±0.0433(b)(BC) | 4.49±0.1450(a)(C) | 5.56±0.1716(b)(D) | 7.89±0.0233(b)(E) | 8.63±0.0503(b)(F) |
| **C5-PC** | 4.32±0.0458(c)(A) | 4.63±0.1278(c)(A) | 5.71±0.0985(c)(B) | 6.64±0.0862(b)(C) | 7.23±0.0788(c)(D) | 8.15±0.1386(b)(E) | 9.13±0.0233(a)(F) |
| **PC-P** | 8.64±0.1795(d)(A) | 9.23±0.1179(d)(B) | 9.69±0.0985(d)(B) | 10.28±0.1178(c)(C) | 11.43±0.0985(d)(D) | 12.43±0.0173(c)(E) | 12.68±0.0219(c)(E) |
| **P2+PC** | 9.21±0.16.50(e)(A) | 9.53±0.0994(de)(A) | 9.46±0.1193(d)(A) | 10.50±0.2074(cd)(B) | 10.93±0.0318(e)(B) | 12.73±0.0265(cd)(C) | 13.08±0.0493(d)(C) |
| **P5+PC** | 9.59±0.0674(e)(A) | 9.81±0.0504(e)(A) | 10.46±0.1193(e)(B) | 11.20±0.1563(d)(C) | 11.60±0.0717(d)(C) | 12.92±0.0393(d)(D) | 13.47±0.0265(e)(D) |

C: control sample (without plasma treatment and phycocyanin pigment); PC-P: sample treated with phycocyanin pigment but without plasma; P2-PC: plasma-treated sample for 2 min without phycocyanin pigment; P5-PC: plasma-treated sample for 5 min without phycocyanin pigment; P2+PC: plasma-treated sample for 2 min with phycocyanin pigment; P5+PC: plasma-treated sample for 5 min with phycocyanin pigment. Different small and capital letters indicate significant differences in the columns and rows, respectively (p < 0.05). All data are expressed as mean ± SEM (n = 3). Data were analyzed using one-way ANOVA followed by Tukey’s post hoc test (p < 0.05).
