# Supplementary material for: Combined effects of gliding-arc plasma and C-phycocyanin on antioxidant activity and shelf-life extension of rainbow trout (Oncorhynchus mykiss) fillets
Source: PLoS One. 2025 Nov 20;20(11):e0336896. doi: 10.1371/journal.pone.0336896 (PMC12633869; doi:10.1371/journal.pone.0336896)
Supplement: S6 Table — C: control sample (without plasma treatment and phycocyanin pigment); PC-P: sample treated with phycocyanin pigment but without plasma; P2-PC: plasma-treated sample for 2 min without phycocyanin pigment; P5-PC: plasma-treated sample for 5 min without phycocyanin pigment; P2 + PC: plasma-treated sample for 2 min with phycocyanin pigment; P5 + PC: plasma-treated sample for 5 min with phycocyanin pigment. Different small and capital letters indicate significant differences in the columns and rows, respectively (p < 0.05). All data are expressed as mean ± SEM (n = 3). Data were analyzed using one-way ANOVA followed by Tukey’s post hoc test (p < 0.05). (DOCX) [file pone.0336896.s010.docx]

**Table S6.** Mean b* of *Oncorhynchus mykiss* fillets treated with GAP and PCP during storage at 4°C for 18 days.

| **b*** | **Day1** | **Day3** | **Day6** | **Day9** | **Day12** | **Day15** | **Day18** |
| --- | --- | --- | --- | --- | --- | --- | --- |
| **C** | 2.08±0.0557(a)(A) | 2.46±0.1093(a)(A) | 3.54±0.0726(a)(B) | 4.56±0.0940(a)(C) | 5.65±0.2924(a)(D) | 6.79±0.0953(a)(E) | 9.65±0.0437(a)(F) |
| **C2-PC** | 2.49±0.0233(b)(A) | 2.92±0.0404(ab)(A) | 3.94±0.0601(a)(B) | 4.51±0.0928(a)(B) | 5.46±0.1968(a)(C) | 6.74±0.3684(a)(D) | 8.69±0.1245(b)(E) |
| **C5-PC** | 3.01±0.0252(c)(A) | 3.43±0.0953(b)(AB) | 3.82±0.1405(a)(B) | 4.52±0.1179(a)(C) | 5.34±0.1157(a)(D) | 6.57±0.0757(a)(E) | 7.98±0.1159(b)(F) |
| **PC-P** | 21.85±0.0954(d)(A) | 21.78±0.3299(c)(A) | 22.78±0.1994(b)(A) | 23.45±0.2167(b)(AB) | 24.99±0.8031(b)(BC) | 25.55±0.3522(b)(CD) | 27.07±0.4491(c)(D) |
| **P2+PC** | 23.39±0.1398(e)(A) | 23.70±0.1069(d)(A) | 24.46±0.1411(c)(B) | 24.99±0.0726(c)(B) | 26.10±0.3053(bc)(C) | 27.70±0.0867(c)(D) | 28.00±0.0115(c)(D) |
| **P5+PC** | 24.22±0.0393(f)(A) | 25.51±0.0764(e)(B) | 26.29±0.0318(d)(C) | 26.92±0.0379(d)(D) | 27.21±0.0451(c)(D) | 27.74±0.1186(c)(E) | 27.96±0.0569(c)(E) |

C: control sample (without plasma treatment and phycocyanin pigment); PC-P: sample treated with phycocyanin pigment but without plasma; P2-PC: plasma-treated sample for 2 min without phycocyanin pigment; P5-PC: plasma-treated sample for 5 min without phycocyanin pigment; P2+PC: plasma-treated sample for 2 min with phycocyanin pigment; P5+PC: plasma-treated sample for 5 min with phycocyanin pigment. Different small and capital letters indicate significant differences in the columns and rows, respectively (p < 0.05). All data are expressed as mean ± SEM (n = 3). Data were analyzed using one-way ANOVA followed by Tukey’s post hoc test (p < 0.05).
