# Supplementary material for: Combined effects of gliding-arc plasma and C-phycocyanin on antioxidant activity and shelf-life extension of rainbow trout (Oncorhynchus mykiss) fillets
Source: PLoS One. 2025 Nov 20;20(11):e0336896. doi: 10.1371/journal.pone.0336896 (PMC12633869; doi:10.1371/journal.pone.0336896)
Supplement: S7 Table — C: control sample (without plasma treatment and phycocyanin pigment); PC-P: sample treated with phycocyanin pigment but without plasma; P2-PC: plasma-treated sample for 2 min without phycocyanin pigment; P5-PC: plasma-treated sample for 5 min without phycocyanin pigment; P2 + PC: plasma-treated sample for 2 min with phycocyanin pigment; P5 + PC: plasma-treated sample for 5 min with phycocyanin pigment. Different small and capital letters indicate significant differences in the columns and rows, respectively (p < 0.05). All data are expressed as mean ± SEM (n = 3). Data were analyzed using one-way ANOVA followed by Tukey’s post hoc test (p < 0.05). (DOCX) [file pone.0336896.s011.docx]

**Table S7**. Mean L***** of *Oncorhynchus mykiss* fillets treated with GAP and PCP during storage at 4°C for 18 days.

| **L*** | **Day1** | **Day3** | **Day6** | **Day9** | **Day12** | **Day15** | **Day18** |
| --- | --- | --- | --- | --- | --- | --- | --- |
| **C** | 70.23±0.1328(a)(A) | 70.10±0.0656(a)(A) | 69.20±0.1266(a)(B) | 67.14±0.1646(a)(C) | 65.06±0.0667(a)(D) | 63.81±0.0318(a)(E) | 61.82±0.0874(a)(F) |
| **C2-PC** | 69.62±0.1170(ab)(A) | 69.01±0.1626(b)(A) | 68.00±0.1291(b)(B) | 67.23±0.2346(a)(C) | 65.36±0.0825(b)(D) | 64.40±0.1290(b)(E) | 63.77±0.0872(b)(E) |
| **C5-PC** | 69.01±0.0808(b)(A) | 68.25±0.0328(c)(B) | 68.06±0.1419(b)(BC) | 67.50±0.2696(a)(C) | 66.69±0.0775(c)(D) | 65.06±0.0088(c)(E) | 64.80±0.0819(c)(E) |
| **PC-P** | 67.16±0.0694(c)(A) | 66.72±0.2572(d)(AB) | 66.26±0.0590(c)(B) | 65.12±0.0470(b)(C) | 64.54±0.0233(d)(C) | 63.78±0.1917(a)(D) | 62.55±0.1245(d)(E) |
| **P2+PC** | 66.48±0.0961(d)(A) | 66.05±0.0757(e)(B) | 66.12±0.0570(c)(AB) | 65.72±0.0570(bc)(B) | 65.22±0.929(b)(C) | 64.19±0.0736(ab)(D) | 63.34±0.1235(b)(E) |
| **P5+PC** | 65.55±0.2396(e)(A) | 64.99±0.0590(f)(AB) | 65.06±0.1235(d)(AB) | 64.55±0.0745(c)(BC) | 64.13±0.0657(f)(CD) | 63.86±0.0203(a)(D) | 62.73±0.0933(d)(E) |

C: control sample (without plasma treatment and phycocyanin pigment); PC-P: sample treated with phycocyanin pigment but without plasma; P2-PC: plasma-treated sample for 2 min without phycocyanin pigment; P5-PC: plasma-treated sample for 5 min without phycocyanin pigment; P2+PC: plasma-treated sample for 2 min with phycocyanin pigment; P5+PC: plasma-treated sample for 5 min with phycocyanin pigment. Different small and capital letters indicate significant differences in the columns and rows, respectively (p < 0.05). All data are expressed as mean ± SEM (n = 3). Data were analyzed using one-way ANOVA followed by Tukey’s post hoc test (p < 0.05).
