# Supplementary material for: Combined effects of gliding-arc plasma and C-phycocyanin on antioxidant activity and shelf-life extension of rainbow trout (Oncorhynchus mykiss) fillets
Source: PLoS One. 2025 Nov 20;20(11):e0336896. doi: 10.1371/journal.pone.0336896 (PMC12633869; doi:10.1371/journal.pone.0336896)
Supplement: S8 Table — C: control sample (without plasma treatment and phycocyanin pigment); PC-P: sample treated with phycocyanin pigment but without plasma; P2-PC: plasma-treated sample for 2 min without phycocyanin pigment; P5-PC: plasma-treated sample for 5 min without phycocyanin pigment; P2 + PC: plasma-treated sample for 2 min with phycocyanin pigment; P5 + PC: plasma-treated sample for 5 min with phycocyanin pigment. Different small and capital letters indicate significant differences in the columns and rows, respectively (p < 0.05). All data are expressed as mean ± SEM (n = 3). Data were analyzed using one-way ANOVA followed by Tukey’s post hoc test (p < 0.05). (DOCX) [file pone.0336896.s012.docx]

**Table S8.** Mean ABTS of *Oncorhynchus mykiss* fillets treated with GAP and PCP during storage at 4°C for 18 days.

| **ABTS** | **Day1** | **Day3** | **Day6** | **Day9** | **Day12** | **Day15** | **Day18** |
| --- | --- | --- | --- | --- | --- | --- | --- |
| **C** | 4.44±0.0444(a)(A) | 4.65±0.0633(a)(A) | 4.93±0.1109(a)(A) | 5.36±0.0214(a)(A) | 7.48±0.0000(a)(B) | 12.79±0.4710(a)(C) | 12.29±0.1661(a)(C) |
| **P2-PC** | 4.39±0.0604(a)(AB) | 4.54±0.0719(a)(A) | 4.05±0.0122(b)(B) | 5.11±0.0943(a)(C) | 5.48±0.0296(b)(D) | 5.70±0.1500(b)(D) | 8.31±0.0000(b)(E) |
| **P5-PC** | 4.40±0.1258(a)(AB) | 4.51±0.1208(a)(AB) | 3.12±0.0442(c)(C) | 4.19±0.1285(b)(A) | 4.69±0.2337(c)(AB) | 4.88±0.1836(b)(B) | 6.56±0.0647(c)(D) |
| **PC-P** | 1.50±0.0000(b)(A) | 1.81±0.0952(b)(AB) | 2.03±0.1562(d)(BC) | 2.19±0.1578(c)(BC) | 2.45±0.0297(d)(CD) | 2.50±0.0487(c)(CD) | 2.78±0.1235(d)(D) |
| **P2+PC** | 1.39±0.0474(b)(A) | 1.44±0.0421(b)(A) | 1.53±0.0675(e)(A) | 1.73±0.0488(d)(AB) | 1.89±0.0699(e)(BC) | 2.33±0.0910(c)(C) | 2.46±0.1124(d)(D) |
| **P5+PC** | 1.39±0.0411(b)(A) | 1.43±0.0583(b)(A) | 1.50±0.0291(e)(A) | 1.59±0.0272(d)(AB) | 1.79±0.0457(e)(BC) | 2.11±0.0841(c)(C) | 2.30±0.0880(d)(D) |

C: control sample (without plasma treatment and phycocyanin pigment); PC-P: sample treated with phycocyanin pigment but without plasma; P2-PC: plasma-treated sample for 2 min without phycocyanin pigment; P5-PC: plasma-treated sample for 5 min without phycocyanin pigment; P2+PC: plasma-treated sample for 2 min with phycocyanin pigment; P5+PC: plasma-treated sample for 5 min with phycocyanin pigment. Different small and capital letters indicate significant differences in the columns and rows, respectively (p < 0.05). All data are expressed as mean ± SEM (n = 3). Data were analyzed using one-way ANOVA followed by Tukey’s post hoc test (p < 0.05).
