# Supplementary material for: Combined effects of gliding-arc plasma and C-phycocyanin on antioxidant activity and shelf-life extension of rainbow trout (Oncorhynchus mykiss) fillets
Source: PLoS One. 2025 Nov 20;20(11):e0336896. doi: 10.1371/journal.pone.0336896 (PMC12633869; doi:10.1371/journal.pone.0336896)
Supplement: S9 Table — C: control sample (without plasma treatment and phycocyanin pigment); PC-P: sample treated with phycocyanin pigment but without plasma; P2-PC: plasma-treated sample for 2 min without phycocyanin pigment; P5-PC: plasma-treated sample for 5 min without phycocyanin pigment; P2 + PC: plasma-treated sample for 2 min with phycocyanin pigment; P5 + PC: plasma-treated sample for 5 min with phycocyanin pigment. Different small and capital letters indicate significant differences in the columns and rows, respectively (p < 0.05). All data are expressed as mean ± SEM (n = 3).Data were analyzed using one-way ANOVA followed by Tukey’s post hoc test (p < 0.05). (DOCX) [file pone.0336896.s013.docx]

**Table S9.** Mean FRAP of *Oncorhynchus mykiss* fillets treated with GAP and PCP during storage at 4°C for 18 days.

| **FRAP** | **Day1** | **Day3** | **Day6** | **Day9** | **Day12** | **Day15** | **Day18** |
| --- | --- | --- | --- | --- | --- | --- | --- |
| **C** | 20.01±0.0000(a)(A) | 19.11±0.1490(a)(B) | 17.09±0.2820(a)(C) | 14.72±0.1290(a)(C) | 14.03±0.0430(a)(D) | 13.17±0.0745(a)(E) | 11.75±0.2686(a)(F) |
| **P2-PC** | 20.40±0.1490(ab)(A) | 19.93±0.1551(a)(A) | 18.42±0.1138(a)(B) | 16.31±0.1138(ab)(C) | 15.11±0.1971(b)(D) | 14.16±0.3011(a)(E) | 13.43±0.0745(b)(E) |
| **P5-PC** | 21.00±0.1138(b)(A) | 20.14±0.1490(a)(B) | 19.67±0.1551(a)(B) | 17.22±0.1875(b)(C) | 16.92±0.1971(c)(C) | 15.84±0.1138(b)(D) | 15.20±0.0430(c)(D) |
| **PC-P** | 32.14±0.2686(c)(A) | 30.98±0.1490(b)(A) | 27.24±0.2264(b)(B) | 26.29±0.2066(c)(BC) | 25.05±0.0745(d)(BC) | 22.89±0.7350(c)(C) | 18.46±0.0745(d)(D) |
| **P2+PC** | 34.64±0.2276(d)(A) | 33.73±0.2276(c)(A) | 32.01±0.1490(c)(B) | 30.98±0.1290(d)(C) | 28.27±0.2235(e)(D) | 25.78±0.1138(d)(E) | 23.50±0.2235(e)(F) |
| **P5+PC** | 35.88±0.1490(e)(A) | 34.81±0.5641(c)(A) | 32.87±0.7041(c)(B) | 32.18±0.2616(d)(BC) | 30.94±0.1875(f)(C) | 26.72±0.1971(d)(D) | 25.30±0.1971(f)(D) |

C: control sample (without plasma treatment and phycocyanin pigment); PC-P: sample treated with phycocyanin pigment but without plasma; P2-PC: plasma-treated sample for 2 min without phycocyanin pigment; P5-PC: plasma-treated sample for 5 min without phycocyanin pigment; P2+PC: plasma-treated sample for 2 min with phycocyanin pigment; P5+PC: plasma-treated sample for 5 min with phycocyanin pigment. Different small and capital letters indicate significant differences in the columns and rows, respectively (p < 0.05). All data are expressed as mean ± SEM (n = 3).Data were analyzed using one-way ANOVA followed by Tukey’s post hoc test (p < 0.05).
